# A MAP Kinase Dependent Feedback Mechanism Controls Rho1 GTPase and Actin Distribution in Yeast

**DOI:** 10.1371/journal.pone.0006089

**Published:** 2009-06-30

**Authors:** Shuguang Guo, Xiaoyun Shen, Gonghong Yan, Dongzhu Ma, Xiaochun Bai, Suoping Li, Yu Jiang

**Affiliations:** 1 Department of Pharmacology, University of Pittsburgh School of Medicine, Pittsburgh, Pennsylvania, United States of America; 2 Department of Bioscience and Biotechnology, Dalian University of Technology, Dalian, Liaoning, People's Republic of China; 3 College of Life Science, Henan University, Kaifeng, People's Republic of China; Duke University Medical Centre, United States of America

## Abstract

In the yeast *Saccharomyces cerevisiae* the guanosine triphosphatase (GTPase) Rho1 controls actin polarization and cell wall expansion. When cells are exposed to various environmental stresses that perturb the cell wall, Rho1 activates Pkc1, a mammalian Protein Kinase C homologue, and Mpk1, a mitogen activated protein kinase (MAPK), resulting in actin depolarization and cell wall remodeling. In this study, we demonstrate a novel feedback loop in this Rho1-mediated Pkc1-MAPK pathway that involves regulation of Rom2, the guanine nucleotide exchange factor of Rho1, by Mpk1, the end kinase of the pathway. This previously unrecognized Mpk1-depedent feedback is a critical step in regulating Rho1 function. Activation of this feedback mechanism is responsible for redistribution of Rom2 and cell wall synthesis activity from the bud to cell periphery under stress conditions. It is also required for terminating Rho1 activity toward the Pkc1-MAPK pathway and for repolarizing actin cytoskeleton and restoring growth after the stressed cells become adapted.

## Introduction

Cells of the yeast *Saccharomyces cerevisiae* grow by a unique process called budding, during which the growth of a cell is restricted at its bud [1], [2]. The polarized growth is maintained by a highly asymmetric distribution of the actin cytoskeleton, which channels transport vesicles to the site of growth for cell wall expansion. Actin distribution in yeast cells is a dynamic process that undergoes depolarization and repolarization during cell cycle or when cells are under stressful conditions [3]. This dynamic process is regulated by a family of small guanosine triphosphatases (GTPase), including Rho1, Cdc42, and several other Rho-type GTPases. Rho1 is the major GTPase involved in maintaining the polarized actin distribution and cell wall expansion during bud growth [4], [5].

Rho1 elicits its function through several distinct effectors, among which are Fks1 and Pkc1 [4], [6], [7]. Fks1 is the catalytic subunit of β-1,3-glucan synthase that catalyzes the synthesis of β-1,3-linked glucan, a major structural component of the yeast cell wall [8]. Pkc1 is a homologue of mammalian Protein Kinase C that controls a mitogen activated protein kinase (MAPK) signaling module composed of Bck1 (MAPKKK), Mkk1 and Mkk2 (MAPKK), and Mpk1/Slt2 (MAPK) [9]. Activation of these two effectors by Rho1 results in changes in actin distribution and cell wall synthesis activity, which are essential for cell survival under stress conditions. Like other small GTPases, the activity of Rho1 depends on its guanine nucleotide binding states. It is active in GTP bound state and inactive in GDP bound state. The guanine nucleotide binding state of Rho1 is reciprocally regulated by its guanine nucleotide exchange factors (GEF) and GTPase activating proteins (GAP) [4]. Several exchange factors of Rho1 have been identified, including two homologous proteins, Rom1 and Rom2, as well as a distantly related protein, Tus1 [10], [11]. Among these proteins, Rom2 is the major GEF for Rho1 that is responsible for relaying signals from cell surface to Rho1 for its activation [10], [12]. Rom2 associates with the plasma membrane through its pleckstrin homology (PH) domain that binds phospholipids as well as its interaction with several integrin-like cell surface proteins, including Wsc1 and Mid2. It has been suggested that these cell surface proteins function as stress sensors to activate Rom2 in response to stresses that alter cell wall integrity [13]–[15]. During normal growth, Rom2 is co-localized with Rho1 at the bud, the site of growth [14], [16]. In response to environmental stresses, such as elevated temperature, both proteins redistribute to cell periphery, where they function together with β-1,3-glucan synthase to remodel the cell wall [13], [17], [18].

The Rho1 mediated Pkc1-MAPK pathway is involved in controlling actin distribution and cell wall expansion [19]. During normal growth, a basal activity of the pathway is required for maintaining the polarized distribution of actin and new wall synthesis. Reduction in its signaling activity often results in randomized actin distribution and a weak cell wall, which causes cell lysis defect at elevated temperature [20], [21]. Activation of this pathway occurs during bud emergence and in response to various stresses that perturb the cell wall [12], [22], [23]. Analysis of heat stress induced activation of the pathway reveals that the activation is a transient process in that the activity of the pathway returns to basal level following a short activation period despite the persistence of the heat stress [11], [22]. The transient activation of the pathway leads to temporary changes in a series of cellular events, including depolarization of actin, redistribution of Rom2 and Rho1 from the bud to cell periphery, a delay in G2/M transition and an increase in transcription of genes involved in cell wall synthesis [17], [24]–[26]. Collaboratively, these events result in cessation of polarized growth and changes in cell wall structure, which allow the affected cells to become adapted to the insult [17], [24]. Despite this, it remains unclear how these events are organized in an orderly manner. In the present study, we identify a novel feedback mechanism in the Rho1 mediated Pkc1-MAPK pathway, in which the end kinase of the pathway, Mpk1, controls the upstream activity through Rom2, the nucleotide exchange factor of Rho1. Our results suggest that this Mpk1 dependent feedback mechanism plays a critical role in coordinating cellular responses to environmental stresses.

## Results

### Rom2 undergoes Mpk1 dependent phosphorylation upon heat stress

**Figure 1:**
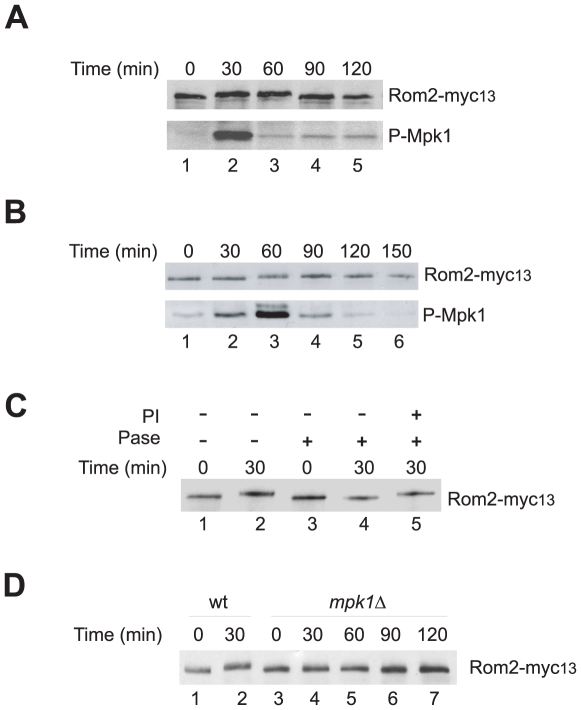
Stress induced Rom2 phosphorylation is Mpk1 dependent. Exponentially growing cells (Y974) were shifted from 23°C to 38°C (A. or treated with rapamycin at 23°C (B. for indicated duration. Cells were collected and lysed. The expressed Rom2-myc_13_ and the levels of phospho-Mpk1 in the lysates were detected by western blotting. C. Wild cells expressing Rom2-myc_13_ (Y974) were grown to early log phase at 23°C and shifted to 38°C. Aliquots of cells were removed at the indicated time points after the shift and lysed. Rom2-myc_13_ was immunopurified with anti-Myc antibody and treated for 1 hr with alkaline phosphatase (Pase+) in the presence (PI+) or absence (PI−) of 20 mM of *β*-glycerophosphate. D. Exponentially growing wild type (Y974) and its isogenic *mpk1*Δ cells expressing Rom2-myc_13_ (Y973) were shifted from 23°C to 38°C. Aliquots of cells were removed at the indicated time points and lysed. The expressed Rom2-myc_13_ in the lysates was detected by western blotting.

In response to mild heat stress the Rho1 mediated Pkc1-MAPK pathway undergoes a transient activation, which culminates at a short duration of an increase in Mpk1 activity [11]. As previous reported [27] and shown in Fig. 1A, the increase in Mpk1 activity was manifested by a rise in the levels of the dual phosphorylation at Thr190/Tyr192 located in the kinase activation loop of Mpk1 (Fig. 1A, lower panel). Interestingly, accompanying the Mpk1 activation was a transient decrease in the mobility of Rom2 on SDS PAGE, which appeared coincidently with Mpk1 activation, and disappeared following Mpk1 inactivation (Fig. 1A, upper panel). The heat induced change in the mobility of Rom2 was also observed with cells treated with cycloheximide, which blocked new protein synthesis (Fig. S1). This observation suggests that the mobility change of Rom2 does not require production of new proteins. To determine whether the mobility shift of Rom2 under heat stress is a common response to an activated Pkc1-MAPK pathway, we treated cells with rapamycin, a drug that is known to stimulate the pathway by inducing starvation responses [28], [29]. As shown in Fig. 1B (upper panel), a similar decrease in Rom2 mobility was detected upon the drug treatment. In comparison with that induced by heat stress, the drug induced change in Rom2 mobility lasted longer, which correlated with an extended duration of Mpk1 activation. Interestingly, while Mpk1 activation appeared 30 min after the drug treatment (lower panel), Rom2 mobility did not change until 60 min later (upper panel). This observation suggests that Mpk1 activation takes place prior to Rom2 mobility shift, implying that the latter might be a consequence rather than a cause for Mpk1 activation.

To determine whether the shift in Rom2 mobility was caused by phosphorylation, we immunopurified Rom2 from heat stressed cells and examined its mobility by SDS PAGE after treating it with alkaline phosphatase. As shown in Fig. 1C, the shift of Rom2 induced by the heat stress was eliminated by the phosphatase treatment (compare lanes 2–4), suggesting that the shift was caused by phosphorylation.

Since the stress induced Mpk1 activation precedes Rom2 phosphorylation, we tested whether Rom2 phosphorylation is dependent on Mpk1 by monitoring the mobility of Rom2 from *mpk1*deletion cells. As shown in Fig. 1D, heat stress failed to cause a change in the mobility of Rom2 in the mutant cells (lanes 3–7), which was in contrast to that in wild type cells (lanes 1 and 2). Since >85% of the *mpk1* deletion cells remained viable at the end of the heat treatment (Fig. S2), the absence of Rom2 mobility shift was unlikely due to cell lysis defect of the mutant cells. These observations suggest that the heat induced Rom2 phosphorylation is dependent on Mpk1.

**Figure 2:**
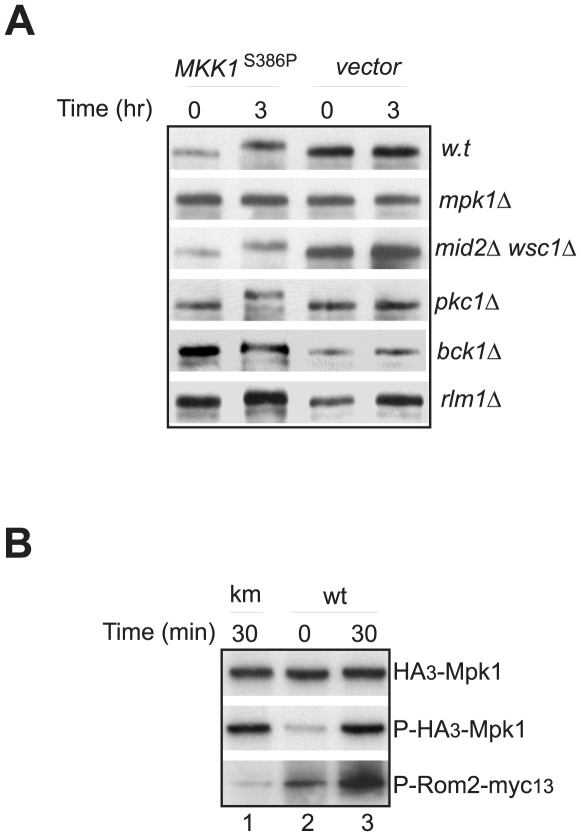
Mpk1 phosphorylates Rom2. A. Yeast cells expressing Myc tagged Rom2, including those of wild type (Y1243), *mpk1*Δ (Y1272)^*^, *wsc1*Δ *mid2*Δ (Y1273)^*^, *pkc1*Δ (Y1274)^*^, *bck1*Δ (Y1275)^*^ and *rlm1*Δ (Y1276) were transformed with *GAL1*p-*MKK1* ^S386P^ or a control vector. Transformed cells were grown overnight in medium containing 2% raffinose to exponential phase, followed by addition of galactose to a final concentration of 1%. An aliquot of cells was collected before or 3 hr after the addition of galactose. Cells were lysed and lysates were analyzed by western blotting with anti-Myc antibody. B. Cells expressing HA_3_-*MPK1* (Y1054) or HA_3_-*mpk1* ^K54R^ (Y1052) were grown to exponential phase at 23°C and shifted to 38°C. Cells were collected at the indicated time points after the shift. HA tagged Mpk1 proteins were immunopurified and incubated with Rom2-myc_13_ in the presence of ATPγ^32^P. The phosphorylation levels of Rom2-myc_13_ by HA_3_-Mpk1 were monitored by the incorporation of ^32^P into the protein (lower panel). The levels of the Mpk1 protein (upper panel) and phospho-Mpk1 (middle panel) were determined by western blotting. Note: * these strains are not isogenic to the wild type strain (Y1243).

Given that Mpk1 is the end kinase in the Pkc1-MAPK pathway, the above observations also raised the possibility that Mpk1 may phosphorylate Rom2 directly. To confirm the notion, we first determined whether overproduction of Mkk1^S386P^ was able to cause Rom2 phosphorylation. Mkk1^S386P^ is an active mutant of Mkk1, a MAPKK in yeast. When overexpressed, it activates Mpk1 independent of upstream signals of the Pkc1-MAPK pathway [30]. As shown in Fig. 2A, Mkk1^S386P^ overproduction induced Rom2 phosphorylation in wild type cells but not in cells deleted of *MPK1*. This finding suggests that *MKK1* ^S386P^ promotes Rom2 phosphorylation through Mpk1. The Mpk1 dependent phosphorylation was also observed in cells deleted of both *WSC1* and *MID2*, indicating that these upstream activators of Rom2 are not required for the phosphorylation. Similarly, deletion of *PKC1* or *BCK1*, which encode two upstream kinases of Mkk1 [9], did not affect the phosphorylation, nor did the deletion of the gene for the transcription factor Rlm1, a target of Mpk1 in transcription [30]. These findings demonstrate that activation of Mpk1 alone is sufficient for Rom2 phosphorylation, supporting the notion that Mpk1 is directly involved in regulation of Rom2.

To further confirm that Mpk1 phosphorylates Rom2 directly, we assessed the ability of Mpk1 to phosphorylate Rom2 using an *in vitro* kinase assay. We found that immunopurified Mpk1 was able to phosphorylate Rom2, and the kinase activity was greatly enhanced when Mpk1 was purified from heat-stressed cells (Fig. 2B, lower panel, compare lanes 2 and 3), which correlated with the heat induced Mpk1 activation (middle panel). In contrast, a kinase inactive mutant of Mpk1 (Mpk1^K54R^) was virtually unable to phosphorylate Rom2 (lane 1). This finding demonstrates that Mpk1 is capable of directly phosphorylating Rom2.

Collectively, the above results strongly suggest that Rom2 is a direct target of Mpk1, thus revealing the existence of a feedback control loop in the Rho1 mediated Pkc1-MAPK pathway, through which the end kinase of the pathway regulates its upstream signaling activity. In the following experiments, we investigated the physiologic significance of this novel feedback control loop.

### Mpk1 is required for Rom2 redistribution from the bud to cell periphery

**Figure 3:**
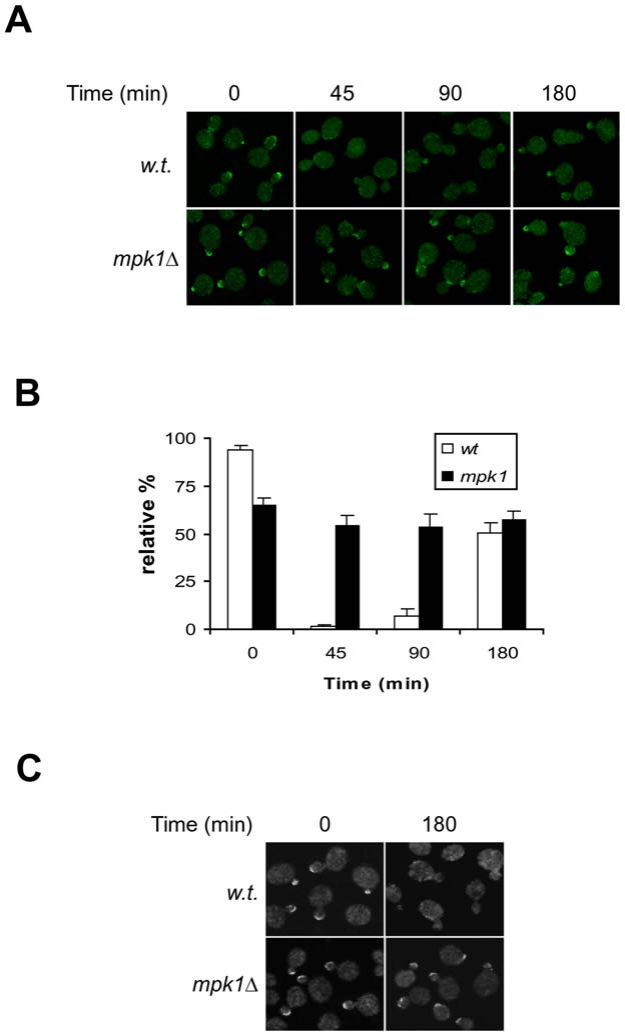
Rom2 undergoes Mpk1 dependent redistribution in response to heat stress. A. Wild type (Y1038) and its isogenic *mpk1*Δ cells (Y1037) expressing *ROM2*-*GFP* were grown to early log phase at 23°C and shifted to 38°C. The localization of Rom2-GFP at the indicated time points after the shift was examined by confocal microscopy. B. The relative percentage of small- and mid-size budded cells showing bud staining of Rom2-GFP at the indicated time points shown in A was quantified. The small- and mid sized buds were defined as those with a cross section bud diameter less than two-thirds of that of the mother cell. Data shown are average of three independent experiments with ≥200 cells counted for each experiment. C. Wild type (Y1038) and *mpk1* deletion (Y1037) cells expressing *ROM2-GFP* and *GAL1*p-*MKK1* ^S386P^ were grown to exponential phase in medium containing raffinose (2%) followed by adding 1% of galactose. At the indicated time points after the addition of galactose, cells were harvested, fixed and imaged by confocal microscopy.

Under normal growth conditions, Rom2 is localized to the bud of actively growing budded cells, where its effector Rho1 resides, but is displaced from the site upon heat stress [13], [14]. We thus determined whether Mpk1 is responsible for this displacement. As shown in Fig. 3A, in wild type cells Rom2 was localized at the bud under normal growth condition, but was quickly displaced from the site in response to heat stress (upper row). In the *mpk1* deletion cells grown under normal condition, Rom2 was also found at the bud, although the percentage of small- or mid-size budded cells displaying bud staining was lower than that of the wild type cells (Fig. 3B). Despite this, the bud localization of Rom2 in the mutant cells was largely unaffected by heat stress (Fig. 3A, lower row), suggesting that Mpk1 is required for displacement of Rom2 from the site. Interestingly, the heat induced displacement of Rom2 in wild type cells was a transient process. Rom2 relocated back to the bud after 90 min despite the persistence of the heat stress, which coincided with Rom2 dephosphorylation following Mpk1 inactivation (Fig. 1A). This observation suggests that the Mpk1 dependent phosphorylation may dictate the localization of Rom2.

To further confirm that heat induced Rom2 relocation is caused by Mpk1 activation, we overexpressed *MKK1* ^S386P^ in both wild type and *mpk1* deletion cells. As shown in Fig. 3C, overexpression of *MKK1* ^S386P^ caused redistribution of Rom2 in wild type cells but not in the mutant ones, suggesting that Mpk1 activation alone is sufficient for Rom2 redistribution.

### Redistribution of Rho1 and Fks1 from the bud to cell periphery does not require Mpk1

**Figure 4:**
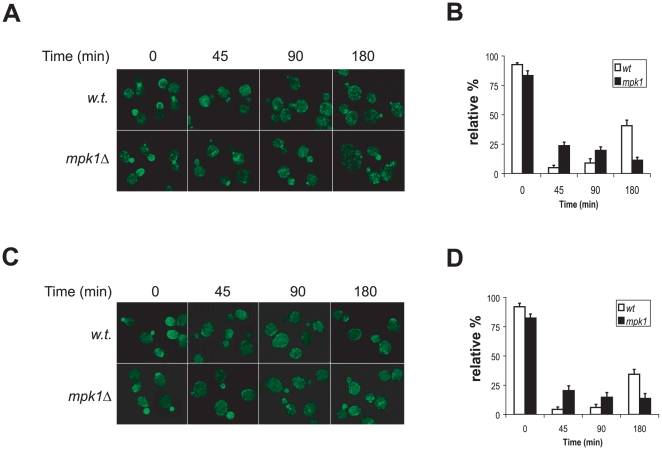
Heat induced redistribution of Rho1 and Fks1 does not require Mpk1. A. Wild type (Y1128) and its isogenic *mpk1*Δ cells (Y1129) expressing HA_3_-*RHO1* were grown to early log phase at 23°C and shifted to 38°C. The localization of HA_3_-Rho1 at the indicated time points after the shift was examined using confocal microscopy. B. The relative percentage of small- and mid-size budded cells showing bud staining of HA_3_-Rho1 at the indicated time points shown in A was quantified. Data shown are average of three independent experiments with ≥200 cells counted for each experiment. C. Wild type (Y1125) and its isogenic *mpk1*Δ cells (Y1126) expressing *FKS1*-GFP were grown to early log phase at 23°C and shifted to 38°C. The localization of Fks1-GFP at the indicated time points after the shift was examined using confocal microscopy. D. The relative percentage of small- and mid-size budded cells showing bud staining of Fks1-GFP at the indicated time points shown in C was quantified. Data shown are average of three independent experiments with ≥200 cells counted for each experiment.

In addition to Rom2, Rho1 and its effector in cell wall synthesis, Fks1, also reside at the bud and undergo stress induced redistribution in a way similar to that of Rom2 [17], [18]. We thus examined whether the redistribution of these two proteins under stress conditions was also controlled by Mpk1. As shown in Fig. 4, we found that upon heat stress Rho1 was displaced from the bud in wild type cells and those deleted of *MPK1* (Figs. 4A and 4B). Similarly, Fks1 was also dislocated in both cell types (Figs. 4C and 4D). These findings suggest that Mpk1 is dispensable for the stress induced redistribution of Rho1 and Fks1. Nevertheless, the displacement of these two proteins in the mutant cells did not appear to be as effective as in wild type cells, since in a significant portion of the small- and mid-size budded cells a small amount of the proteins remained at the bud (Fig. 4). This observation indicates that Mpk1 may play an indirect role in regulating the redistribution of Rho1 and Fks1, likely through controlling Rom2 relocation. Collectively, these findings suggest that Rom2 is the primary target of the Mpk1 dependent feedback mechanism.

### Mpk1 is required for inactivation of the Rho1 mediated Pkc1-MAPK pathway

**Figure 5:**
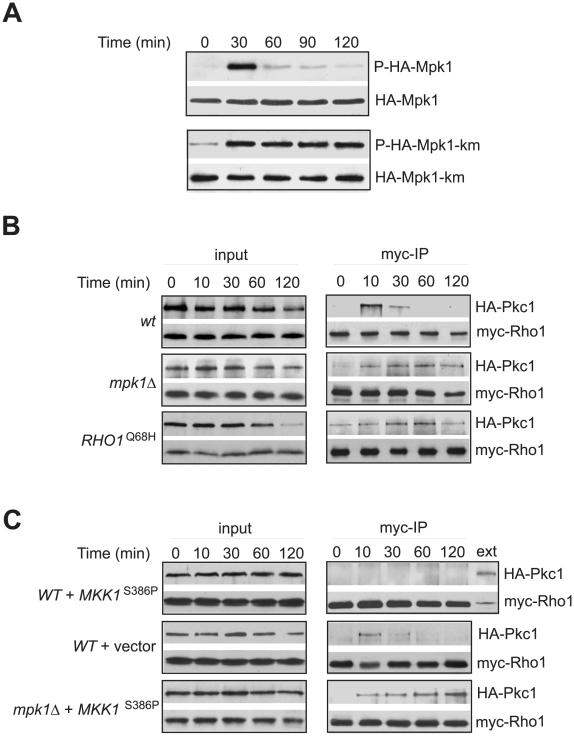
Mpk1 is required for inactivation of Rho1. A. The *mpk1*Δ cells (Y972) expressing either HA tagged wild type *MPK1* (upper panels) or the *mpk1* ^K54R^ mutant gene (lower panels) were grown at 23°C to early log phrase and shifted to 38°C. Aliquots of cells were collected at the indicated time points after the shift. The levels of phosphorylated wild type (P-HA-Mpk1) and kinase mutant Mpk1 (P-HA-Mpk1-km) were detected using anti-phospho MAPK antibody (1^st^ and 3^rd^ panels). The levels of both wild type (HA-Mpk1) and kinase mutant Mpk1 (HA-Mpk1-km) were detected using anti-HA antibody (2^nd^ and 4^th^ panels). B. Exponentially growing yeast cells of isogenic strains, Y1277 (wt), Y1281 (*mpk1*Δ) and Y1278 (*RHO1* ^Q68H^), were shifted from 23°C to 38°C. At indicated time points after the shift, aliquots of cells were removed and lysed. Cell lysates were precipitated with anti-Myc antibody and precipitates were analyzed by western blotting for the presence of Myc_3_-Rho1 and Pkc1-HA_3_. C. Wild type (Y1277) and *mpk1*Δ (Y1281) cells expressing *MKK1* ^S386P^ or control vector were grown at 23°C to exponential phase in medium containing 2% raffinose. The expression of *MKK1* ^S386P^ was induced by addition of galactose to a final concentration of 1%. Upon induction for 3 hr, cells were shifted to 38°C. Aliquots of cells were removed at the indicated time points after the shift and lysed for immunoprecipitation as in B.

The Mpk1 dependent redistribution of Rom2 reveals the existence of a feedback mechanism by which the end kinase of the Pkc1-MAPK pathway affects the upstream signaling activity. One potential outcome of the feedback is that the Mpk1 dependent redistribution of Rom2 deprives Rho1 activity from Pkc1, and consequently, leads to Pkc1 inactivation. Should this be the case, inactivation of Mpk1 would result in a sustained activation of the Pkc1-MAPK pathway. To test this notion, we examined the signaling activity of the pathway in the absence of Mpk1. Accordingly, we expressed a wild type or a kinase inactive mutant of Mpk1 (Mpk1^K54R^) in *mpk1* deletion cells and monitored the dual phosphorylation levels at Thr190/Tyr192 of the ectopically expressed proteins. Because Mpk1^K54R^ contains a Lys to Arg point mutation in its kinase domain that abrogates its kinase activity [31], this mutant is able to receive Mkk1/Mkk2 directed phosphorylation at Thr190/Tyr192 but is unable to become activated, thus incapable of initiating the feedback. On the other hand, wild type Mpk1 is able to set off the feedback upon Mkk1/Mkk2 directed phosphorylation. As shown in Fig. 5A, in cells expressing wild type Mpk1 heat stress induced a short duration of increase in the phosphorylation levels at Thr190/Tyr192 of the expressed protein, indicating that the activation of pathway was transient. In contrast, in cells expressing the mutant Mpk1, the same treatment caused a prolonged increase in the phosphorylation, indicating that the activation of the pathway was sustained.

Because *mpk1* deletion cells are defective in several cellular processes, including G2/M transition, cell wall integrity and transcription, it is possible that the sustained activation of the Pkc1-MAPK pathway in the mutant cells was caused indirectly by malfunctions in these processes. To rule out this possibility, we examined the activity of the pathway in cells bearing similar defects. We found that heat induced activation of the pathway was effectively terminated in cells arrested at G2/M by nocodazole treatment, blocked for cell wall synthesis or deleted of the major transcriptional target of Mpk1, Rlm1 (Fig. S3). These results indicate that the sustained activation of the Pkc1-MAPK pathway in *mpk1* deletion cells is unlikely to be an indirect consequence of Mpk1 inactivation.

### Mpk1 negatively regulates Rho1

To determine that Mpk1 downregulates the Pkc1-MAPK pathway by inhibiting Rho1 activity toward Pkc1, we first investigated whether in the absence of Mpk1, Rho1 undergoes a sustained activation upon heat stress. Previous studies have shown that Rho1 interacts with Pkc1 only in active GTP-bound form [32], [33]. We thus exploited the ability of Rho1 to interact with Pkc1 as an indication for Rho1 activation. In wild type cells heat stress induced a transient interaction between Rho1 and Pkc1, but in *mpk1* deletion cells the same treatment caused a prolonged interaction between Rho1 and Pkc1. As a control, we found that Rho1^Q68H^, an active mutant of Rho1 [34], exhibited a constitutive interaction with Pkc1 (Fig. 5B, lower panels). These findings demonstrated that heat induced Rho1 activation was sustained in the absence of Mpk1.

To further confirm the negatively role of Mpk1 in Rho1 regulation, we examined whether *MKK1* ^S386P^ overproduction, which drives Mpk1 activation, was able to repress heat induced Rho1 activation. As shown in Fig. 5C, we found that heat failed to induce the association of Rho1 with Pkc1 in wild type cells expressing *MKK1* ^S386P^, but caused a prolonged association of Rho1 with Pkc1 in *mpk1* deletion cells expressing the same gene. These observations demonstrate that high Mpk1 activity prevents Rho1 activation.

### The Mpk1-Rom2 feedback loop mediates the lethal effect of *MKK1* ^S386P^

**Figure 6:**
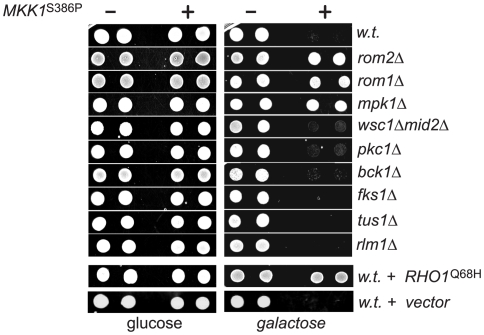
The lethal effect of *MKK1* ^S386P^ overexpression is caused by Mpk1 dependent inhibition of Rho1. Yeast cells expressing *GAL1*p-*MKK1* ^S386P^ (+) or a control vector (-) were spotted onto plates containing 1 M sorbitol with either 2% of glucose or 2% of raffinose plus 1% of galactose. Plates were imaged after incubation at 30°C for two days. Strains used were wt (Y661), *rom2*Δ (Y741), *rom1*Δ (AAY265)*, *mpk1*Δ (Y972)*, *wsc1*Δ *mid2*Δ (Y1214)*, *pkc1*Δ (D376)*, *bck1*Δ (Y1177)*, *fks1*Δ (Y1216)*, *tus*1Δ (1203)* and *rlm1*Δ (Y1221). Note: * these strains are not isogenic to Y661.

The existence of the Mpk1-Rom2 feedback loop in the Rho1 mediated Pkc1-MAPK pathway predicts that a sustained activation of Mpk1 would inhibit Rho1 and Pkc1 dependent processes. Coincidently, it was previously shown that overexpression of the *MKK1* ^S386P^ mutant inhibited cell growth through a Mpk1 dependent mechanism [30]. It is thus possible that the lethality associated with *MKK1* ^S386P^ overproduction was caused by inhibition of Rho1 through the Mpk1 dependent feedback loop. To test this possibility, we first determined whether expressing *RHO1* ^Q68H^ is able to rescue the *MKK1* ^S386P^ induced growth arrest. We found that expressing the active but not wild type *RHO1* permitted cell growth in the presence of *MKK1* ^S386P^ overproduction (Fig. 6A), suggesting that the downregulation of Rho1 is likely to be the cause for *MKK1* ^S386P^ induced growth arrest. To further determine whether the Mpk1 mediated feedback loop is involved in the growth arrest, we examined the effect of *MKK1* ^S386P^ overexpression in cells deleted of *ROM2* or *MPK1*. As shown in Fig. 6B, whereas overexpression of *MKK1* ^S386P^ inhibited the growth of wild type cells, it failed to do so to cells depleted of *ROM2* or *MPK1*. This result suggests that the inhibitory effect of *MKK1* ^S386P^ is mediated through the Mpk1 dependent feedback mechanism. Furthermore, we determined whether other components of the Rho1-mediated Pkc1-MAPK pathway are involved. Like wild type cells, those deleted of both *WSC1* and *MID2*, *PKC1*, *BCK1*, *FKS1* and *RLM1* remained sensitive to *MKK1* ^S386P^ overexpression, suggesting these gene products are not required for the inhibitory activity of *MKK1* ^S386P^. Interestingly, deletion of *ROM1*, which encodes a GEF of Rho1, rendered cells resistant to *MKK1* ^S386P^ overexpression, while deletion of *TUS1,* encoding another GEF of Rho1, did not. This observation suggests that Rom1, but not Tus1, functions similarly to Rom2 in the feedback loop. Collectively, the above findings demonstrate that other components of the Rho1 mediated PKC1-MAPK pathway are not involved in the inhibitory feedback loop, further endorsing the notion that the loop constitutes a direct phosphorylation of Rom2, and possibly, of Rom1, by Mpk1.

### Downregulation of Pkc1 is required for actin repolarization in *mpk1* deletion cells

**Figure 7:**
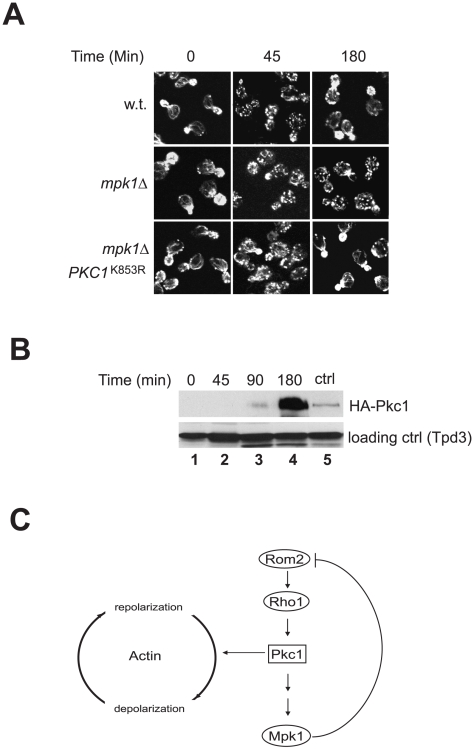
Downregulation of Pkc1 is required for actin repolarization in *mpk1*Δ cells. A. Wild type (Y062) and its isogenic *mpk1*Δ cells (Y972) expressing *GAL1*p-*PKC1* ^K853R^ or a control vector were grown in medium containing 2% of raffinose at 23°C to early log phase and shifted to 38°C. Galactose was added to the medium to a final concentration of 1% immediately following the shift. Aliquots of cells were collected, fixed at the indicated time points, stained with fluor-phalloidin and visualized by confocal microscopy. B. Expression levels of Pkc1^K853R^ in *mpk1*Δ cells (Y972) at the indicated time points after the shift. The level of an HA tagged endogenous Pkc1 in wild type cells was shown as a control (ctrl). C. A model for the action of Mpk1 in the Rho1 mediated Pkc1-MAPK pathway and actin distribution (see text for detail).

In response to heat stress, actin undergoes a transient depolarization followed by repolarization. While the depolarization depends on Pkc1 activation, the repolarization requires Mpk1. In the absence of Mpk1, depolarized actin is unable to repolarize and remains disorganized [17]. In light of the finding that Mpk1 is required for terminating the upstream signaling activity of Pkc1, it is possible that the requirement of Mpk1 for actin repolarization reflects a need for Mpk1 to downregulate Pkc1. To test this possibility, we examined whether inhibition of Pkc1 was able to repolarize actin in the absence of Mpk1. As shown in Fig. 7A, we found that actin became depolarized in both wild type and *mpk1* deletion cells upon exposure to heat stress for 45 min. Within 3 hr, actin repolarized in wild type cells but remained depolarized in the mutant cells. These observations are consistent with the previous finding [17]. However, when a dominant negative *PKC1* mutant, *PKC1* ^K853R^ [7], was overexpressed (Fig. 7B), it permitted actin repolarization in the mutant cells (Fig. 7A, lower row). This observation indicates that downregulation of Pkc1 is required for actin repolarization, thus supporting a notion that Pkc1 activity controls both depolarization and repolarization of actin (Fig. 7C).

## Discussion

The Rho1 GTPase mediated Pkc1-MAPK pathway in yeast cells regulates actin organization and cell wall expansion during normal growth or under stress conditions [3], [5]. The signaling process of the pathway is believed to involve a series of sequential reactions that culminate at the activation of Mpk1, the end kinase of the pathway [9]. In the present study, we uncovered a Mpk1 dependent feedback loop that is an integral part of the signaling processes of the pathway. This novel feedback loop involves phosphorylation of Rom2, the upstream activator of Rho1, by the end kinase of the pathway, Mpk1, leading to redistribution of Rom2 from the bud to cell periphery and termination of the Pkc1-MAPK cascade. This finding indicates that the signaling mechanisms of Rho1 are more complicated than previously appreciated.

The redistribution of Rom2, Rho1 and Fks1 from the bud to cell periphery upon heat stress is believed to shift cell wall synthesis activity to cell periphery, where repairing or remodeling of cell wall is required for the affected cells to withstand the insult [13], [17]. Because Rom2 fails to relocate to cell periphery in the absence of Mpk1 (Fig. 3A), it is conceivable that Rom2 redistribution is not caused by signals from upstream but by a feedback from Mpk1. Consistent with this notion, we found that activation of Mpk1 by overexpression of *MKK1* ^S386P^ was sufficient to direct Rom2 to cell periphery (Fig. 3C). While Mpk1 controls redistribution of Rom2, it does not affect that of Rho1 and Fks1 (Figs. 3 and 4). In the absence of Mpk1, Rho1 and Fks1 are still able to relocate from the bud tip to cell periphery. However, they are unable to become active at the site, because Rom2, the activator of Rho1, is absent. As a consequence, the affected cells lost the ability to repair or remodel cell wall at cell periphery, which may contribute to the cell lysis defect associated with *mpk1* deletion cells.

The sustained signaling activity of the Pkc1-MAPK pathway in the absence of Mpk1 indicates that the Mpk1 dependent feedback is required to terminate the signaling activity of the pathway (Fig. 5). It is possible that the Mpk1 dependent feedback inhibits the Pkc1-MAPK pathway by downregulating the GEF activity of Rom2. However, a downregulated Rom2 activity would jeopardize its function for cell wall repairing or remodeling at cell periphery, leading to cell lysis defect under stress conditions. Since this scenario does not happen to wild type cells under mild heat stress, we consider the possibility unlikely. On the other hand, given that Pkc1 resides at sites of polarized growth during cell cycle, such as the bud tip and bud neck [35], it is possible that the redistribution of Rom2 and Rho1 from the bud tip to cell periphery deprives of the upstream signaling activity from Pkc1, causing its inactivation. In the absence of Mpk1, Rom2, together with some Rho1 remained at the bud, may keep Pkc1 active. In this regard, Mpk1 acts as a negative regulator of the pathway.

The negative role of Mpk1 also explains the lethality associated with *MKK1* ^S386P^ overexpression. As the direct activator of Mpk1, *MKK1* ^S386P^ overexpression is expected to stimulate and uphold Mpk1 activity, resulting in a sustained Rom2 phosphorylation. It may be assumed that the phosphorylated Rom2 causes downregulation of Rho1 function, because an active allele of *RHO1* alleviates the lethal effect of *MKK1* ^S386P^ overexpression (Fig. 6). Nevertheless, it appears that the downregulation in Rho1 activity is not caused simply by Rom2 inactivation, because inactivation of Rom2 or Rom1 by genetic deletion abolishes the lethal effect of *MKK1* ^S386P^ overexpression rather than exaggerates it. The requirement for Rom1 and Rom2 for the lethal activity of *MKK1* ^S386P^ overexpression indicates that these factors, upon phosphorylation, acquire an activity that negatively regulates Rho1. It remains unclear how this negative regulation is achieved. It is possible that the Mpk1 dependent phosphorylation of Rom2 may prevent Rho1 and Fks1 from re-locating back to the bud, and consequently block bud growth. Further study is needed to determine the underlying mechanism.

In response to heat stress actin undergoes a transient depolarization and repolarization process. It has been found that the upstream components of the pathway, including Rom2, Rho1 and Pkc1, are required for depolarization, but Mpk1 is required for actin repolarization. These observations have led to the suggestion that Pkc1 controls actin distribution through both Mpk1 dependent and independent mechanisms [17]. Our findings suggest that the role of Mpk1 in actin repolarization process is to terminate Pkc1 activity by removing its upstream activators, which supports a notion that Pkc1 activity dictates both actin depolarization and repolarization. It is likely that Pkc1 activation promotes and sustains actin depolarization and its inactivation allows actin repolarization (Fig. 7C).

The existence of a Mpk1 dependent feedback control as an integral part of the Rho1 mediated Pkc1-MAPK pathway suggests that there are two sequential signaling activities that propagate through the pathway when cells are exposed to adverse conditions. In the first phase, Rho1 is activated by Rom2 in response to stresses, which in turn promotes Pkc1 and Mpk1 activation. Pkc1 activation leads to actin depolarization, and consequently inhibits bud growth by disrupting vesicular transport. Upon activation, Mpk1 elicits at least three different functions. First, it increases transcription of genes involved in cell wall synthesis [25]. Second, it inactivates Cdc28 via a checkpoint mechanism that causes a delay in cell cycle progression [24], [26]. Finally, it initiates the second phase of signaling by phosphorylating Rom2 and directing cell wall synthesis activity toward cell periphery (Figs. 1 and 3). In doing so, it removes the upstream activators from Pkc1, causing its inactivation and actin repolarization. Conceivably, the Mpk1 dependent feedback loop also leads to its own inactivation, which may allow Rom2 and Rho1 to relocate back to the bud so that growth may resume. Hence, Mpk1, the end kinase of the Rho1 mediated Pkc1-MAPK pathway, acts as a switch to control the signaling activity of Rho1 and Pkc1 and to coordinate cellular responses to environmental stresses.

## Materials and Methods

### Media and reagents

Yeast cells were normally grown in YP medium containing 1% yeast extract and 2% peptone or synthetic complete media lacking an appropriate amino acid for selection. All media normally contain 2% glucose as the carbon source unless indicated otherwise. Rapamycin and FK506 were purchased from LC laboratories (Woburn, MA), and Nocodazole and cycloheximide from Sigma. Anti-phospho-MAPK (Thr202/Tyr204) antibody was purchased from Cell Signaling (Beverly, MA), and anti-HA and myc antibodies from Roche (Indianapolis, IN).

### Yeast strains and plasmids

Yeast strains used in this study are listed in Table S1 of the Supplementary Materials S1. Plasmid expressing a dominant negative *PKC1* gene under control of the *GAL1* promoter, *GAL1*p-*PKC1* ^K853R^, was a generous gift from David Levin [7]. Plasmids pNV7-*MKK1* ^S386P^ expressing an active allele of *MKK1* (*MKK1* ^S386P^) under the *GAL1* promoter, the control vector pNV7 and the *rlm1Δ::LEU2* construct were kindly provided by Kunihiro Mutsumoto [30]. To construct HA_3_-*MPK1*-pRS314, a 2.2 kb fragment containing the *MPK1* gene was amplified by PCR from yeast genomic DNA and cloned into pRS314. A sequence cassette containing triple tandem repeats of HA epitope was inserted at the second codon the *MPK1* gene. HA_3_-*MPK1*-pRS424 was made by cloning the HA_3_-*MPK1* gene into pRS424. The HA_3_-*mpk1* ^K54R^ kinase mutant was generated by site directed mutagenesis using QuikChange kit from Strategene. To construct Myc_3_-*RHO1-*pRS406, a 1.4 kb fragment containing *RHO1* gene was amplified by PCR and cloned into pRS406, and a sequence cassette containing triple tandem repeats of Myc epitope was inserted at the second codon of the *RHO1* gene. Myc_3_-*RHO1* ^Q68H^ was created by site directed mutagenesis. A 5′ end truncated Myc epitope-tagged *ROM2* gene (*rom2*Δ5′-myc_13_) was constructed by cloning a SacI-BamHI PCR fragment containing the last 658 base pairs of the *ROM2* gene into pRS406. A sequence cassette containing 13 tandem myc epitopes (myc_13_) and an *ADH1* termination sequence was then inserted into the BamHI site that was placed immediately before the stop codon. Plasmid *rom2*Δ5′-myc_13_-pRS404 was created by cloning the truncated myc tagged *ROM2* gene into pRS404. A 5′ truncated HA tagged *PKC1* gene (*pkc1*Δ5′-HA_3_) was generated by cloning a 2.1 kb fragment containing the 3′ region of *PKC1* (from 1301 upstream of the stop codon to 795 bp after) into pRS406 and followed by inserting a triple HA sequence cassette immediately before the stop codon. The epitope-tagged genes were used to replace their endogenous versions by two step-gene replacement method [36].

### Fluorescence Microscopy

For analysis of actin distribution, cells were fixed with formaldehyde and stained with Alexa-conjugated phalloidin (Molecular Probes) as described before [37]. Localization studies of HA_3_-Rho1 were performed as previously described [17]. For localization studies of GFP tagged Rom2 and Fks1, cells were collected, fixed in methanol at −20°C for 10 min and washed once with acetone, 3× with PBS. After the final wash, cells were resuspended in PBS and visualized by confocal microscopy (Zeiss Pascal 5 M).

### *In vitro* kinase assay

The kinase activity of Mpk1 toward Rom2 was determined using immunopurified epitope tagged Mpk1 and Rom2. To purify Rom2-myc_13_, Y974 cells were grown in YPD medium at 30°C to exponential phase. Cells (2×10^9^) were harvested and lysed with glass beads in 0.5 ml of lysis buffer containing 50 mM Tris-Cl, pH 7.4, 150 mM NaCl, 1 mM DTT, 1 mM EDTA, 1% Triton X-100, 5 mM sodium pyrophosphate, 5 mM *β*-glycerophosphate, 5 mM Na_3_VO_4_ and 1× protease inhibitor cocktail (Roche, Indianapolis, IN). Lysates (10 mg) were incubated for 3 hr with 120 µl of Protein A agarose beads conjugated with anti-myc antibody. Beads were washed 3× with lysis buffer and twice with kinase buffer containing 50 mM Tris-Cl, pH 6.8, 1 mM DTT, 10 mM MgCl_2_, 0.1% Triton X-100. The washed beads were divided into aliquots of 20 µl each and used for kinase reaction. To purify HA_3_-Mpk1, Y972 cells were transformed with plasmid HA_3_-*MPK1*-pRS424 or HA_3_-*mpk1* ^K54R^-pRS424, and transformed cells were grown to exponential phase at 23°C and then shifted to 38°C. Aliquots of cells (5×10^8^) were collected before and 30 min after the shift and lysed in 0.5 ml of lysis buffer. Lysates (3 mg) were precipitated with 20 µl of Protein A agarose bead conjugated with anti-HA antibody. Beads were washed 3× with lysis buffer and twice with kinase buffer. After the final wash, beads were resuspended in 70 µl of kinase buffer containing 5 mM HA epitope peptides (GenScript, Piscataway, NJ) and incubated for 30 min at 30°C. Beads were removed after the incubation by centrifugation. An aliquot of 30 µl of supernatant was incubated with 20 µl of Rom2-myc_13_ bounded beads in the presence of 100 µM ATP, 1 µCi ATPγ ^32^P at 30°C for 30 min. The reaction was terminated by adding 15 µl of 5× SDS sample buffer followed by boiling for 5 min. Samples were subjected to SDS PAGE and transferred to nitrocellular membrane. Phosphorylated Rom2 was visualized by radioautography and the amount of HA_3_-Mpk1 and phospho-Mpk1 (Thr190/Tyr192) in each reaction was determined by western blotting.

### Co-immunoprecipitation

Yeast cells expressing *PKC1*-HA_3_ and Myc_3_-*RHO1* were grown overnight at 23°C to early log phase and shifted to 38°C. At each of the indicated time points after the shift, an aliquot of cells (2×10^9^) was transferred to a centrifuge tube filled with ice and collected by centrifugation at 500 g for 5 min. After washing once with ice-cold lysis buffer containing 50 mM Tris-Cl, pH 7.4, 100 mM NaCl, 1% Triton X-100, 2 mM DTT, 1 mM PMSF and 1.5× EDTA-free protease inhibitor cocktail (Roche), cells were resuspended in 0.5 ml lysis buffer and lysed with glass beads. Lysate (10 mg) was incubated 2 µg of anti-Myc antibody (9E10) for 2 hrs at 4°C and followed by addition of 15 µl of Protein A beads. After incubation for 1 hr, beads were washed 3× with wash buffer containing 50 mM Tris-Cl, pH 7.4, 300 mM NaCl, 1% Triton X-100, and once with 20 mM Tris-Cl, pH 7.4. After the final wash, beads were resuspended in 60 µl of 2× SDS sample buffer. Upon incubation at 65°C for 10 min, samples were immediately subjected to SDS PAGE and western blot analysis. HRP-linked protein A/G was used to replace secondary antibody for detecting Myc_3_-Rho1 in western blotting.

## Supporting Information

Materials S1 (0.04 MB DOC)

Table S1 (0.09 MB DOC)

Figure S1 Heat induced Rom2 phosphorylation does not require new protein synthesis. Exponentially growing cells (Y974) were shifted from 23°C to 38°C. Cycloheximide was added to the culture to a final concentration of 15 µg/ml at the time of the shift. Aliquots of cells were collected and lysed at the indicated time points after the shift. The expressed Rom2-myc13 (upper row) and the levels of phosphor-Mpk1 (lower row) in the lysates were determined by western blotting.
(1.16 MB EPS)

Figure S2 Viability of mpk1Δ cells under mild heat stress. Exponentially growing mpk1Δ (Y972) cells were shifted from 23°C to 38°C. Aliquots of cells were removed at the indicated time points after the shift and plated on YPD plates containing 1 M sorbitol. Cell viability was determined by the percentage of the plated cells that formed colonies after incubation at 30°C for two days.
(0.86 MB EPS)

Figure S3 Defects in cell wall synthesis, G2/M transition and Rlm1 dependent transcription do not cause sustained activation of the Pkc1-MAPK pathway. Top Panel: Wild type cells (Y1054) were grown to early log phase at 23°C and shifted 38°C. Nocodazole or drug vehicle control (ctrl) was added to the culture to a final concentration of 15 µg/ml at the time of the shift. At the indicated time points after the shift, aliquots of cells were collected and lysed. The levels of HA3-Mpk1 and phospho-Mpk1 in the lysates were determined by western blotting. Middle Panel: The fks1Δ cells (Y1216) expressing HA3-MPK1 were grown to early log phase at 23°C and shifted to 38°C. FK506 or drug vehicle control (ctrl) was added to the culture to a final concentration of 1 µg/ml, which blocked the expression of FKS2 and thus prevented cell wall synthesis in the mutant cell [9], [10]. Cells were collected and processed as described above. Bottom Panel: The rlm1Δ mpk1Δ cells expressing HA3-MPK1 (Y1269) or HA3-mpk1(K54R) (Y1270) were grown to early log phase at 23°C and shifted to 38°C. Cells were collected and processed as described above.
(2.47 MB EPS)
