# Supplementary material for: A MAP Kinase Dependent Feedback Mechanism Controls Rho1 GTPase and Actin Distribution in Yeast
Source: PLoS One. 2009 Jun 30;4(6):e6089. doi: 10.1371/journal.pone.0006089 (PMC2699537; doi:10.1371/journal.pone.0006089)
Supplement: Materials S1 — (0.04 MB DOC) [file pone.0006089.s001.doc]

**Supplementary Materials**

**Yeast strains**

Yeast strains used in this study are listed in Table S1. Strains AAY265 and AAY522 were generously provided by Scott Emr [1], Y741 by Michael Snyder [2], D376 and D2282 by David Levin [3], [4], PA120-3B and TS100-1B by Michael Hall [5], and YOC2439 by Yoshikazu Ohya [6]. Y972, Y973 and Y974 were derivatives of Y062. Y1037 and Y1038 were progenies from a cross between AAY522 and Y972; Y1125 and Y1126 between YOC2439 and Y972; Y1128 and Y1129 between PA120-3B and Y972; Y1201 and Y1203 between TS100-1B and AYY265. Y1214 was generated from D2282 by marker switch. Y1216 was derived from YOC2239. Y1221 was created from Y661 by one-step gene replacement using *rlm1::LEU2* construct. Y1269 was generated from a cross between Y1251 and Y972. Y1272 was derived from Y972, Y1273 from Y1214, Y1274 from D376, Y1275 from Y1201 and Y1276 from Y1221. Strains Y1277, Y1278 and Y1281 were created by replacing the endogenous *PKC1* and *RHO1* with epitope tagged versions of the genes using two-step gene replacement [7].

**References**

1. Audhya A, Emr SD (2002) Stt4 PI 4-kinase localizes to the plasma membrane and functions in the Pkc1-mediated MAP kinase cascade. Dev Cell 2: 593-605.

2. Manning BD, Padmanabha R, Snyder M (1997) The Rho-GEF Rom2p localizes to sites of polarized cell growth and participates in cytoskeletal functions in Saccharomyces cerevisiae. Mol Biol Cell 8: 1829-1844.

3. Levin DE, Bartlett-Heubusch E (1992) Mutants in the S. cerevisiae PKC1 gene display a cell cycle-specific osmotic stability defect. J Cell Biol 116: 1221-1229.

4. Philip B, Levin DE (2001) Wsc1 and Mid2 are cell surface sensors for cell wall integrity signaling that act through Rom2, a guanine nucleotide exchange factor for Rho1. Mol Cell Biol 21: 271-280.

5. Delley PA, Hall MN (1999) Cell wall stress depolarizes cell growth via hyperactivation of RHO1. J Cell Biol 147: 163-174.

6. Utsugi T, Minemura M, Hirata A, Abe M, Watanabe D, et al. (2002) Movement of yeast 1,3-beta-glucan synthase is essential for uniform cell wall synthesis. Genes Cells 7: 1-9.

7. Rothstein R (1991) Targeting, disruption, raplacement, and rescue:intergrative DNA transformation in yeast. Method Enzymol 194: 281-301.

8. Thomas BJ, Rothstein R (1989) Elevated recombination rates in transcriptionally active DNA. Cell 56: 619-630.

9. Garrett-Engele P, Moilanen B, Cyert MS (1995) Calcineurin, the Ca2+/calmodulin-dependent protein phosphatase, is essential in yeast mutants with cell integrity defects and in mutants that lack a functional vacuolar H(+)-ATPase. Mol Cell Biol 15: 4103-4114.

10. Mazur P, Morin N, Baginsky W, el-Sherbeini M, Clemas JA, et al. (1995) Differential expression and function of two homologous subunits of yeast 1,3-beta-D-glucan synthase. Mol Cell Biol 15: 5671-5681.
