# Supplementary material for: A MAP Kinase Dependent Feedback Mechanism Controls Rho1 GTPase and Actin Distribution in Yeast
Source: PLoS One. 2009 Jun 30;4(6):e6089. doi: 10.1371/journal.pone.0006089 (PMC2699537; doi:10.1371/journal.pone.0006089)
Supplement: Table S1 — (0.09 MB DOC) [file pone.0006089.s002.doc]

**Table S1.** Yeast strains used in this study.

| Strain | Genotype | Reference and source |
| --- | --- | --- |
| Y062  Y661^a^ | *MAT*α *his3 leu2 trp1 ura3*  *MAT*a *ura3 his3 trp1 leu2 ade2 (W303-1A)* | Lab stock  [8] |
| Y741 | *MAT*α *rom2::HIS3 ura3 his3 trp1 leu2 lys2 ade2* | [2] |
| Y972 | *MAT*α *mpk1::LEU2 his3 leu2 trp1 ura3* | This study |
| Y973 | *MAT*a *mpk1::LEU2 rom2::ROM2-myc_13_:URA3 his3 leu2 trp1 ura3* | This study |
| Y974 | *MAT*a *rom2::ROM2-13myc_13_:URA3* *his3 leu2 trp1 ura3* | This study |
| Y1037 | *MAT*a *mpk1::LEU2 rom2::ROM2-GFP:HIS3MX6 ura3 his3 leu2 trp1* | This study |
| Y1038 | *MAT*a *rom2::ROM2-GFP:HIS3MX6 ura3 his3 trp1 leu2* | This study |
| Y1052 | *MAT*a *mpk1::LEU2 rom2::ROM2-myc_13_:URA3 his3 leu2 trp1 ura3* [HA_3_*-mpk1^K54R^-*pRS424] | This study |
| Y1054 | *MAT*a *mpk1::LEU2 rom2::ROM2-myc_13_:URA3 his3 leu2 trp1 ura3* [HA_3_*-MPK1-*pRS424] | This study |
| Y1125 | *MAT*a *ade2 his3 lys2 trp1 ura3 fks1::HIS3 ade3::FKS1-GFP:TRP1 fks2::LYS2* | This study |
| Y1126 | *MAT*a *ade2 his3 lys2 trp1 ura3 mpk1::LEU2 fks1::HIS3 ade3::FKS1-GFP:TRP1 fks2::LYS2* | This study |
| Y1128 | *MAT*α *rho1::KanMX4 leu2 ura3 trp1 his3 GAL^+^* [HA_3_*-RHO1-*Ycplac33] | This study |
| Y1129 | *MAT*a *rho1::KanMX4 mpk1::LEU2 leu2 ura3 trp1 his4 GAL^+^* [HA_3_*-RHO1-*Ycplac33] | This study |
| Y1177 | *MAT*a *bck1::KMX his3-∆1 leu2∆0 met15 ∆0 ura3 ∆0* | ATCC |
| Y1201 | *MAT*a *bck1::HIS3 rom2::ROM2-GFP:HIS3MX6 leu2 ura3 his3 rme1* | This study |
| Y1203 | *MAT*α *tus1::KanMX4 leu2 ura3 trp1 his3* | This study |
| Y1214 | *MAT*a *wsc1::LEU2 mid2::TRP1 leu2 ura3 trp1 his4* | This study |
| Y1216 | *MAT*α *fks1::HIS3 his3 trp1 ura3 leu2* | This Study |
| Y1221 ^a^ | *MAT*a *rlm1::LEU2 ura3 his3 trp1 leu2 ade2* | This study |
| Y1243 ^a^ | *MAT*a *rom2::ROM2-myc_13_ ura3 his3 trp1 leu2 ade2* | This study |
| Y1251 ^a^ | *MAT*a *rlm1::LEU2 ura3 his3 trp1 leu2 ade2* [HA_3_*-MPK1-*pRS424] | This study |
| Y1269 ^a^ | *rlm1::LEU2 mpk1::LEU2 rom2::ROM2-myc_13_:URA3* [HA_3_*-MPK1-*pRS424] | This study |
| Y1270 ^a^ | *rlm1::LEU2 mpk1::LEU2 rom2::ROM2-myc_13_:URA3* [HA_3_*-mpk1^K54R^-*pRS424] | This study |
| Y1271 ^a^ | *MAT*a *rom2::ROM2-myc_13_:TRP1 ura3 his3 trp1 leu2 ade2* | This study |
| Y1272 | *MAT*α *mpk1::LEU2 ROM2-myc_13_:TRP1 his3 leu2 trp1 ura3* | This study |
| Y1273 | *MAT*a *wsc1::LEU2 mid2::TRP1 rom2::ROM2-myc_13_:HIS3* | This study |
| Y1274 | *MAT*a *pkc1::LEU2 rom2::ROM2-myc_13_:TRP1 leu2 ura3 trp1 his4* | This study |
| Y1275 | *MAT*a *bck1::HIS3 rom2::ROM2- myc_13_:TRP1 leu2 ura3 his3 rme1* | This study |
| Y1276 ^a^ | *MAT*a *rlm1::LEU2 rom2::ROM2- myc_13_:TRP1 ura3 his3 trp1 leu2 ade2* | This study |
| Y1277 ^a^ | *pkc1:: PKC1-HA_3_ rho1::Myc_3_-RHO1 ura3 his3 leu2 trp1 ade2* | This study |
| Y1278 ^a^ | *MAT*a  *pkc1:: PKC1-HA_3_ rho1:: Myc_3_-RHO1^Q68H^ ura3 his3 leu2 trp1 ade2* | This study |
| Y1281 ^a^ | *MAT*a  *pkc1:: PKC1-HA_3_ rho1::Myc_3_-RHO1 mpk1::LEU2,ura3 his3 leu2 trp1 ade2* | This study |
| AAY265 | *MATα rom1::LEU2 leu2 ura3 his3 trp1 lys2 suc2* | [1] |
| AAY522 | *MAT*α *rom2::ROM2-GFP:HIS3MX6 leu2 ura3 his3 trp1 lys2 suc2* | [1] |
| D376 | *MAT*a  *pkc1::LEU2 leu2 ura3 trp1 his4* | [3] |
| D2282 | *MAT*a *mid2::URA3 wsc1::LEU2 leu2 ura3 trp1 his4* | [4] |
| PA120-3b | *MAT*a *rho1::KanMX4 leu2 ura3 trp1 his4 rme1 GAL^+^* [HA_3_*-RHO1-*Ycplac33] | [5] |
| TS100-1B | *MAT*a *bck1::HIS3 MX6 tus1::KanMX4 leu2 ura3 rme1 trp1* |  |
| YOC2439 | *MAT*a *ade2 his3 lys2 trp1 ura3 fks1::HIS3 fks2::LYS2 ade3::FKS1-GFP:TRP1* | [6] |

^a^ derivatives of Y661
